# A novel homozygous splice-site variant in the *FOCAD* gene causing infantile liver cirrhosis and neutropenia: expanding disease phenotype and successful surgical treatment

**DOI:** 10.3389/fmed.2025.1680857

**Published:** 2026-01-13

**Authors:** Ekaterina Nuzhnaya, Elena Zaklyazminskaya, Viktoriia Zabnenkova, Darya Akimova, Evgeny Tatarsky, Lana Dzik, Andrey Surkov, Natalia Zhurkova, Andrey Filin, Aleksej Metelin, Anna Arakelyan, Viktoriya Savina, Arshak Babayan, Mikhail Skoblov, Natalia Semenova

**Affiliations:** 1 Federal State Budgetary Institution, Research Centre for Medical Genetics, Moscow, Russia; 2 Petrovsky National Research Centre of Surgery, NRCS, The Federal Agency for Scientific Organizations, Moscow, Russia; 3 Pirogov Russian National Research Medical University, Moscow, Russia

**Keywords:** FOCAD, infantile cirrhosis, liver disease, microcephaly, neutropenia

## Abstract

**Background:**

Progressive liver cirrhosis in pediatric patients characterized by clinical and genetic variability. Infantile cirrhosis caused by biallelic variants in the *FOCAD* gene is an extremely rare multi-system disorder leading to the progressive liver dysfunction. A small number of patients with limited survival were described so far, and any new clinical observation can provide a new insight for complete phenotypic spectrum and perspectives on available treatment to meet patient’s needs.

**Methods:**

We performed clinical, instrumental, histological and laboratory evaluation of the family with patient (male, 3 y.o.) with progressive liver cirrhosis and apparently healthy parents. Genetic study was performed using whole-exome sequencing. Validation of the rare variant found on WES and cascade familial screening were performed by capillary Sanger sequencing. Functional validation included RNA analysis from patient-derived fibroblasts and *in silico* protein modeling.

**Results:**

The patient exhibited an expanded phenotype including microcephaly, macrotia and neutropenia. Genetic testing revealed a novel homozygous *FOCAD* splice-site variant NM_001375570.1:c.1455 + 1G > T *FOCAD* (NM_001375570.1):c.1455 + 1G > T affecting RNA splicing with in-frame deletion of 36 nucleotides. Aberrant protein lacks 12 aminoacids (p.Thr475_Val486del) resulting in loss of two conserved *α*-helices. Structural modeling predicted impaired protein stability. At 25 months, the patient underwent a living-donor liver transplantation with a good clinical result, and favorable outcome in 1 year post-liver transplant.

**Conclusion:**

Canonic splice site variant NM_001375570.1:c.1455 + 1G > T *FOCAD* (NM_001375570.1):c.1455 + 1G > T realizes through in-frame deletion of 12 amimoacids (p.Thr475_Val486del) of the FOCAD protein with detectable expression in patient-derived cells. Homozygous carrier of this pathogenic variant exhibits progressive hepatic failure expanded with neutropenia. Liver transplantation had a good long-term result, and can be considered as a promising surgical approach for patients with *FOCAD*-related infantile cirrhosis.

## 1 Introduction

Liver cirrhosis is a major global health burden, ranking as the 11th leading cause of mortality and accounting for over one million deaths annually. When combined with hepatocellular carcinoma, cirrhosis contributes to approximately 3.5% of all global deaths, underscoring its clinical and public health importance (1). While liver disease in adults is well studied, the pediatric cirrhosis, particularly in infancy, remains less explored, and often represents etiologically and clinically different entity.

In infants, cirrhosis most commonly arises from biliary atresia, familial intrahepatic cholestasis syndromes and metabolic disorders, whereas older children are more often affected by autoimmune and genetic conditions such as autoimmune hepatitis, Wilson’s disease, alpha-1-antitrypsin deficiency, and primary sclerosing cholangitis (2, 3). Liver transplantation remains the only curative option for pediatric end-stage liver disease.

A syndromic form of infantile liver cirrhosis associated with biallelic *FOCAD* variants was first reported in 2022, describing a cohort of 14 affected individuals and establishing the disorder as a distinct clinical entity, subsequently annotated in OMIM (MIM #619991) as a liver disease, severe congenital. Notably, immunological manifestations were not described in this initial report (4).

Here, we describe a novel pathogenic splice-site variant in *FOCAD* in an infant with progressive liver cirrhosis and neutropenia. We provide functional validation of its pathogenicity, expand the phenotypic spectrum of *FOCAD*-related disease and present the first long-term survival following successful liver transplantation.

## 2 Materials and methods

### 2.1 Clinical data

The proband was examined at the Research and Counseling Department of the Research Centre for Medical Genetics (RCMG, Moscow), Pirogov Russian National Research Medical University (PRNSMU, Moscow) and at the Petrovsky National Research Centre of Surgery (NRCS, Moscow). Initial evaluation included comprehensive medical history, detailed physical examination and assessment of growth parameters, which were interpreted using standard deviation (SD) scores based on age- and sex-matched normative reference data. Diagnostic work-up comprised a blood tests, abdominal ultrasonography and percutaneous liver biopsy for histological analysis.

Informed consent for participation in the study and publication of clinical data was obtained from the patient’s parents, acting as legal guardians. The study was approved by the Local Ethics Committee of the Research Centre for Medical Genetics (approval number 11/23.11.2021, Moscow, Russia). Written consent for publication of the patient’s photographs was obtained from the parents as legal guardians.

Blood samples from the proband and unaffected parents were collected, and genomic DNA was extracted by standard methods with QIAamp DNA Blood Mini Kit (Qiagen, Hilden, Germany). The WES analysis was carried out using paired-end sequencing (2 × 150 bp) on an IlluminaNextSeq 500 sequencer (Illumina, San Diego, California, U. S.). The library was constructed using the KAPA Hyper Prep Kit (F. Hoffmann-La Roche Ltd., Switzerland). Target enrichment was performed with an IDT xGen® Exome Research Panel v.1 solution capture array (IDT inc., USA), including the coding regions of 19,396 known genes. The detected variants were annotated according to the HGVS nomenclature: https://varnomen.hgvs.org/recommendations/DNA/ (version 21.1.1). The sequencing data were analyzed using the NGS-data-Genome program developed at the Department of Bioinformatics of FSBSI RCMG (registration number 2021662113). Mean coverage was ×81, with 5.8% of fragments with less than ×10 coverage. The following predictive algorithm to analyze the pathogenicity of the variants was used: SpliceAI (James T Robinson, Helga Thorvaldsdottir, Douglass Turner, Jill P Mesirov), igv.js: an embeddable JavaScript implementation of the Integrative Genomics Viewer (IGV), Bioinformatics, Volume 39, Issue 1, January 2023, btac830 (5). Automatic Sanger sequencing was carried out using ABIPrism 3,500 Genetic Analyzer (Applied Biosystems, Foster City, CA, USA) according to the manufacturer’s protocol. Primer sequences were chosen according to the NM_001375570.1: FOCAD_11F: TTGTTGGAAGGAGTGATA; FOCAD_11R: AAGCAGGAATAATCTACATT.

Pathogenicity assessment of the detected variants were classified according to the guidelines for Massive parallel sequencing (MPS) data interpretation (6). Whole exome sequencing (WES) for proband was performed using the facilities and equipment of the “Genome” Shared Resource Centre at the Research Centre for Medical Genetics (Moscow, Russia). Cascade familial screening was performed in Petrovsky NRCS and post-test genetic counseling was performed in RCMG.

### 2.2 RNA analysis

Total RNA was extracted from primary skin fibroblast cultures obtained from the proband and both parents. All cell lines were acquired from and cryopreserved at the Moscow Branch of the All-Russian Collection of Biological Samples of Hereditary Diseases Biobank. RNA extraction was performed using the Extract RNA reagent (Evrogen, Russia), followed by cDNA synthesis with the Reverse Transcription System (Dialat, Russia) in accordance with the manufacturer’s protocol. cDNA integrity was verified through quantitative PCR (qPCR) amplification of the B2M housekeeping gene. Targeted next generation sequencing of RT-PCR product NGS libraries were prepared using the “SG GM” Kit (Raissol) and sequenced on the FASTASeq platform in a paired-end mode (2 × 150 b.p.). The target locus achieved >40,000 × mean read depth across all samples. The raw sequencing data was processed with a custom pipeline based on open-source bioinformatics tools. In brief, the pipeline involved quality control of raw reads using the FastQC tool v0.12.1, followed by read mapping to the hg38 human genome assembly and sorting using STAR 2.7.11b. Splice junctions were visualized using Sashimi plot in IGV.

### 2.3 Structural visualization

The predicted 3D structure of the FOCAD protein (UniProt ID: Q5VW36) was retrieved from AlphaFold DB (accession code: AF-Q5VW36-F1) in PDB format, structural visualization and analysis were performed using the PyMOL Molecular Graphics System (v2.5.8, Schrödinger, LLC) (7, 8).

## 3 Results

### 3.1 Clinical evaluation

The proband was a 1-year-old male born to non-consanguineous parents with no significant family history. Delivery occurred at 40 weeks of gestation, with a birth weight of 2,490 g (−2.49 SD) and a length of 50 cm (−0.52 SD). The APGAR scores were 7 and 8 at 1 and 5 min, respectively. The perinatal period was uneventful and the proband passed the neonatal screening. The first six months of development were unremarkable. However, starting at 6 months of age a delay in weight gain was noted. Motor development was slightly delayed, with rolling over at 6 months and independent sitting achieved at 8 months.

At 8 months of age, the patient was hospitalized due to impaired weight gain and persistent diarrhea. Clinical and laboratory evaluations revealed elevated transaminase levels (more than three times the upper limit of normal), mild conjugated hyperbilirubinemia, dyslipidemia, coagulopathy and neutropenia since the age of 1 year as detailed in Table 1.

**Table 1:** Anthropometric data and blood test results: pre- and post-transplantation.

Parameters	Normal	8 m	1 y 1 m	1 y 4 m	1 y 8 m		2 y 4 m	3 y 3 m
Weight, kg/SD	±2 SD	N/D	8.3/−1.45	N/D	N/D	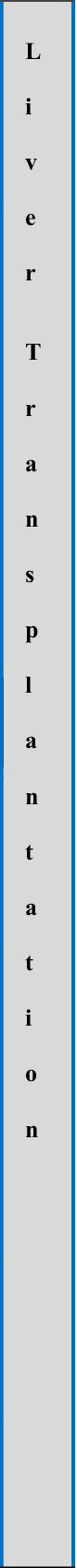	N/D	13/−0.93
Height, sm/SD	±2 SD	N/D	74/ -0.91	N/D	N/D		N/D	93/+0.88
Weight/Height/SD	±2 SD	N/D	−1.40	N/D	N/D		N/D	−0.40
Head circumference, sm/SD	±2 SD	N/D	44/−1.68	N/D	N/D		N/D	46/**−2.62**
Blood test results								
Hemoglobin	**110–140 g/L**	**100**	**83**	**91**	**88**		**102**	**104**
Red blood cells	3.5–4.5 * 10 ^**12**^ **/L**	**3.44**	**3.22**	3.6	3.68		3.67	3.88
White blood cells	6–17.5 *10 ^**9**^ **/L**	8.4	**1.5**	5	4.6		**3.14**	**5.4**
Platelets	160–390 * 10 ^**9**^ **/L**	**107**	**52**	**144**	**117**		**132**	**146**
Total bilirubin	**5–21 mol/L**	5.4	9.5	6.7	**22.8**		11.4	9.6
Direct bilirubin	**< 3.4 mol/L**	**8.3**	3.5	2.1	3.2		2.6	2.5
ALT	**0–40 U/L**	**102.7**	**84**	**76**	**77.9**		41	**50.7**
AST	**0–40 U/L**	**112.3**	**140.2**	**86**	41.3		24	**49**
ALP	**82–383 U/L**	**710.9**	288.8	303	334.1		157	326
GGT	**6–12 m: < 34;** **1–3 y: < 18 U/L**	**40.1**	**36.5**	20.1	17.2		**40**	**35**
AFP	**0.5–50,000 IU/mL**	835.4						
Total protein	**64–83 g/L**	**59.6**	**54.1**	63.4	**55.9**		70	77.5
Albumin	**5–52 g/L**	27.8		30.9	24.2		44.7	35
Total cholesterol	**2.96–5.26 mol/L**	**2.6**	**2.1**	**2.4**	**1.6**		3	**2.1**
LDL-C	**1.5–4.0 mol/L**				**0.790**			
HDL-C	**0.67–1.99 mol/L**				**0.470**			
TG	**0.33–1.12 mol/L**				0.760			
Glucose	**3.3–5.6 mol/L**	**2.2**	4.8		3.8		4.1	3.9
Lactic acid	**0.5–2.5 mol/L**	**6.8**	2.4		**3.6**			
Fibrinogen	**2–3.93 g/L**	**1.7**	**1.81**	2.3	2.26		**1.86**	2.21
PI	**77–138%**	**57.9**	85.5	**74**	**73**			
aPTT	**25.1–36.5 s.**	28	28.2	34.7	**39**		**20.6**	35.3
INR		1.33	1.0	1.12	1.25		0.98	1.24

AFP, alpha-fetoprotein; ALT, alanine aminotransferase; ALP, alkaline phosphatase; aPTT, activated partial thromboplastin time; AST, aspartate aminotransferase; GGT, glutamyltranspeptidase; HDL-C, high-density lipoprotein cholesterol; INR, international normalized ratio; LDL – С, low-density lipoprotein cholesterol; m, months; N/D, no date; PI, prothrombin index; SD, standard deviation; TG, triglycerides; y, years.

Imaging studies demonstrated hepatomegaly and diffuse hepatic changes consistent with cirrhosis. No structural abnormalities were detected on the echocardiogram. Urine screening showed no abnormal bile acids level. Tandem mass spectrometry (MS/MS) analysis of acylcarnitines revealed elevated tyrosine levels, while succinylacetone levels remained normal, suggesting secondary changes in the context of underlying liver cirrhosis.

**Figure 1:**
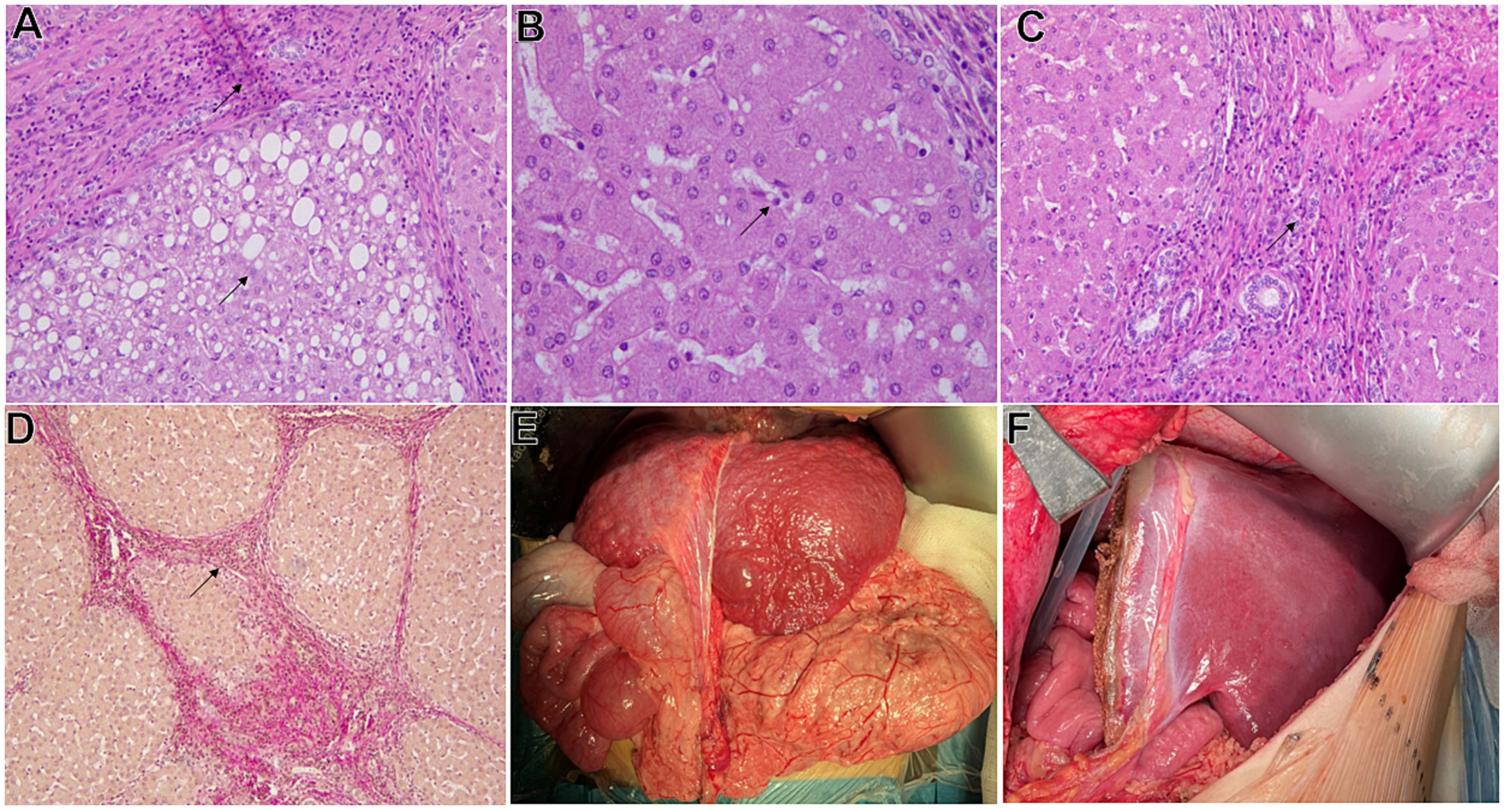
Macroscopic and histopathological characteristics of the liver in a patient. **(А)** A section of liver tissue with fibrosis in the form of connective tissue septa with weak lymphoid infiltration as indicated by the arrow in the image. Macro- and microvesicular fatty degeneration of hepatocytes as indicated by the arrow in the image. Hematoxylin and eosin staining, magnification x200. **(B)** Microvesicular fatty degeneration of hepatocytes as indicated by the arrow in the image. Hematoxylin and eosin staining, magnification x400. **(C)** Bile ducts proliferation in the area of connective tissue septa as indicated by the arrow in the image. Hematoxylin and eosin staining, magnification x200. **(D)** The liver tissue is divided by connective tissue septa into small and large nodes as indicated by the arrow in the image. Van Gieson elastic staining, magnification x100. **(E)** Cirrhotic liver of the patient. Macroscopic overview. **(F)** Graft of the left lateral bisegment of the donor’s liver. General view.

At the age of 2 years a puncture liver biopsy revealed changes indicative of micronodular cirrhosis with focal macro- and microvesicular fatty degeneration of hepatocytes, proliferation of bile ducts and lymphoid infiltration as shown in Figures 1A–D.

Patient underwent a liver transplantation of the lateral bisegment from a living related donor (father) at the age of 2 years and 1 month (Figures 1E,F). The late postoperative period was complicated by the development of small bowel obstruction, necessitating a relaparotomy, abdominal organ revision, and resolution of the obstruction through the division of the antireflux spur and optimal repositioning of the intestinal anastomosis. Four months post-transplant, signs of graft rejection appeared, leading to a liver biopsy and initiation of pulse therapy with methylprednisolone. The patient’s condition stabilized and a repeat biopsy showed no signs of dysfunction. The patient was discharged on a dual immunosuppressive regimen with a progressive dose reduction. No further episodes were observed during follow-up. However, since the age of 2 years and 3 months the patient has exhibited persistent neutropenia which has been managed with granulocyte colony-stimulating factor.

**Figure 2:**
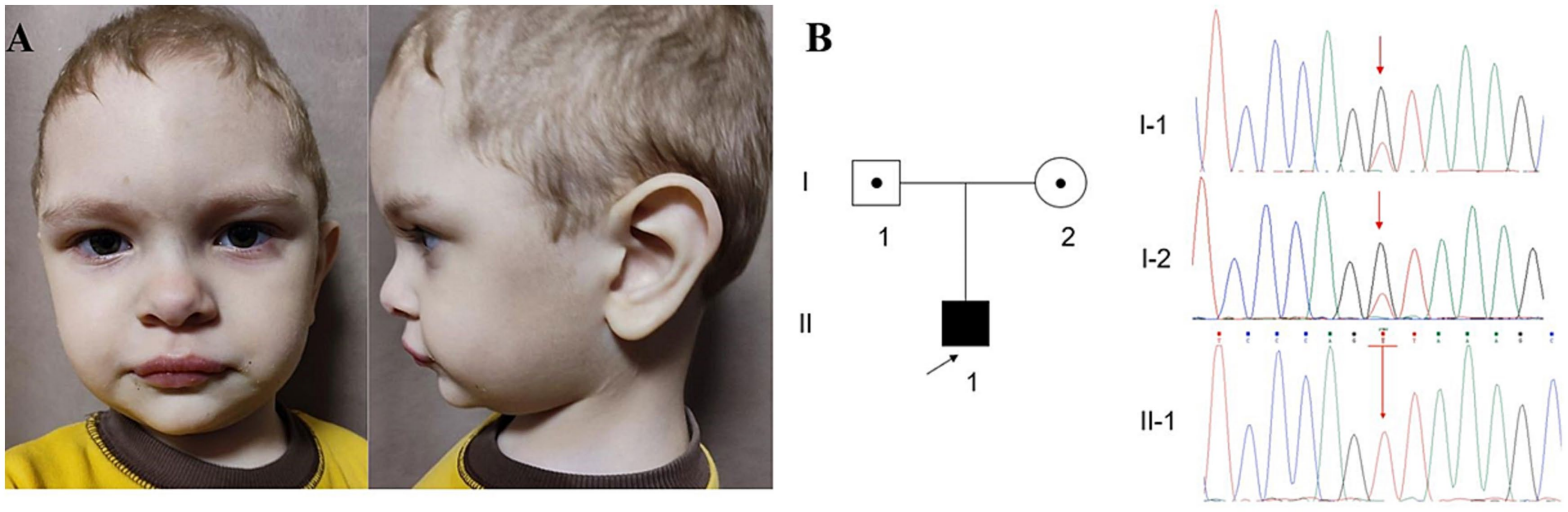
Facial abnormalities, family tree and genetic testing of the proband with the variant in *FOCAD*. **(А)** Facial abnormalities with microcephaly (−2.62 SD) at 3 years 3 months. **(B)** Pedigree showing the affected proband and their unaffected parents. Sanger sequencing results demonstrating c.1455 + 1G > T truncating *FOCAD* variant in the homozygous state in the proband and in the heterozygous state in both parents.

During longitudinal follow-up, the patient’s phenotype became increasingly distinctive with characteristic craniofacial features becoming more apparent by the age of 3 years and 3 months as shown in Figure 2.

Notable dysmorphic features included a round face (HP:0000311), microcephaly (HP:0000252), a prominent metopic ridge (HP:0005487), narrow forehead (HP:0000341), horizontally oriented and long eyebrows (HP:0011228; HP:0004523), broad eyebrows (HP:0011229), deeply set eyes (HP:0000490), epicanthal folds (HP:0000286), a low-hanging columella (HP:0009765), a broad philtrum (HP:0000289), and macrotia (HP:0000400), sparse hair (HP:0008070), thin hair (HP:0002209), single transverse palmar crease (HP:0000954).

The patient is currently 3 years and 3 months old, demonstrates adequate weight gain, and is progressing well in terms of age-appropriate development. Laboratory investigations reveal mild cytolysis with low enzymatic activity, accompanied by mild anemia and neutropenia. The patient is also under regular ophthalmologic follow-up for myopia, endocrinologic monitoring for subclinical hypothyroidism and surgical evaluation for a right-sided inguinoscrotal hernia.

### 3.2 Molecular findings

WES was performed for detecting cause of disease. The only expected deleterious variant was a homozygous NM_001375570.1:c.1455 + 1G > T splice site variant in the intron 11 region of the *FOCAD* gene. The identified nucleotide sequence variant is listed in the control population of the Genome Aggregation Database (gnomAD v4.1.0) with extremely low allele frequency of 0.00004091% as of 12 June 2025. Sanger sequencing confirmed a homozygous state of the variant in patient and in heterozygous states in both parents. According to *in silico* prediction using the SpliceAI tool, the NM_001375570.1:c.1455 + 1G > T variant is expected to abolish the canonical donor splice site and activate a cryptic donor site within intron 11, resulting in a 36-nucleotide (12-amino acid) in-frame deletion.

According to the ACMG/AMP guidelines (6), the NM_001375570.1: c.1455 + 1G > T variant in the *FOCAD* meets criteria PVS1 and PM2. Based on this evidence, it is classified as likely pathogenic and considered the cause of the disease in this individual.

To experimentally validate this prediction, RNA analysis was conducted. Specifically, deep targeted sequencing of the *FOCAD* mRNA region containing the NM_001375570.1: c.1455 + 1G > T variant was performed on samples from the proband and the proband’s parents.

**Figure 3:**
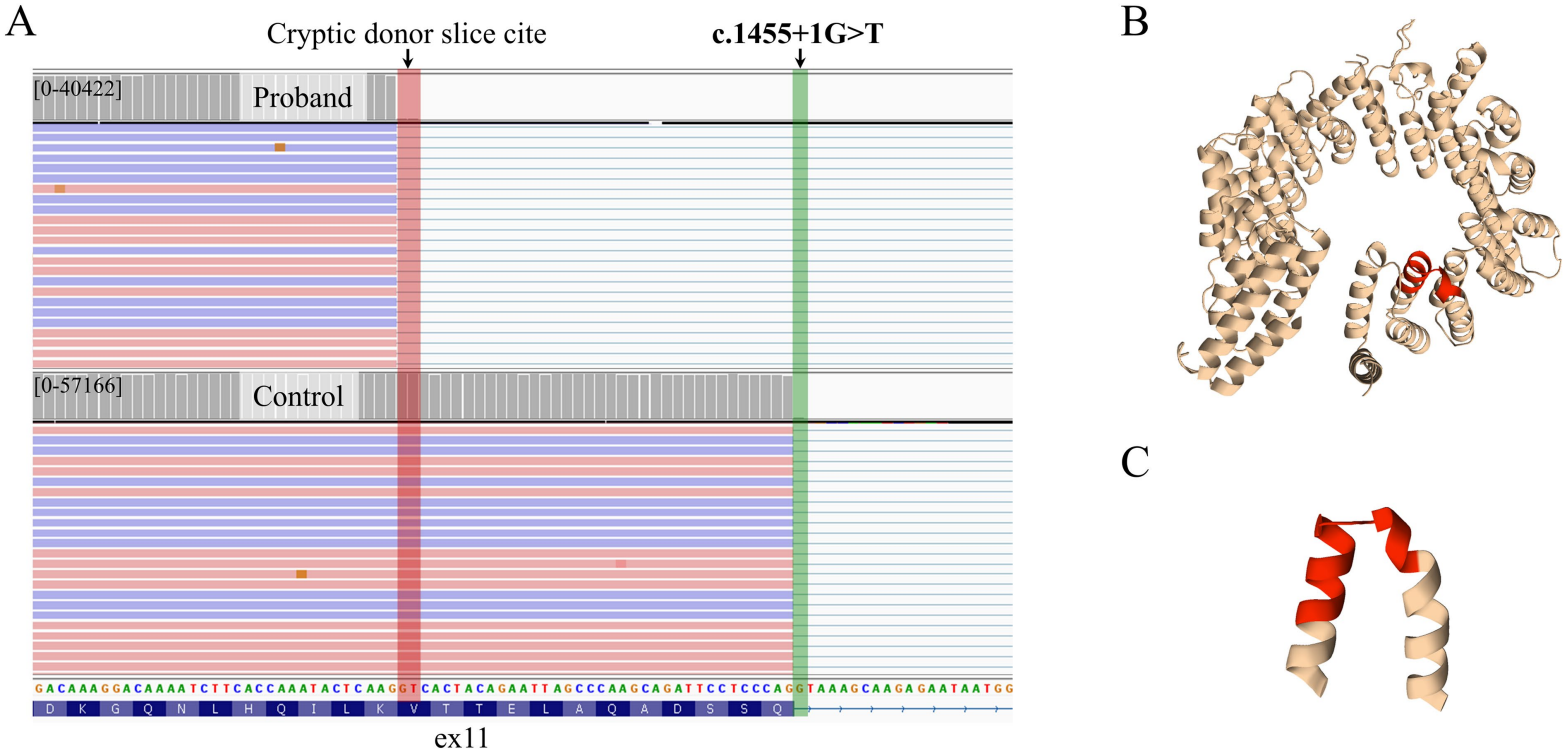
Functional analysis of *FOCAD* variant NM_001375570.1: c.1455 + 1G > T. **(A)** The Sashimi plot demonstrates the aberrant splicing of exon 11 in *FOCAD*, specifically showing the truncation of exon 11 in both the proband and the proband’s mother. **(B)** Predicted 3D conformation of repetitive alpha hairpins region of *FOCAD* as predicted by AlphaFold (v.2). Affected in-frame deletion highlighted in red. **(С)** Close-up view of two affected alpha helices. The deleted region (residues 474–486) is highlighted in red.

The analysis revealed that the proband, homozygous for this variant, expressed only an aberrant transcript isoform with a 36-nucleotide truncation in exon 11 (Figures 3A). Analysis of the targeted cDNA locus in the proband’s parents, who are heterozygous carriers of the c.1455 + 1G > T variant, revealed the presence of both the reference *FOCAD* transcript and transcripts with a 36-nucleotide truncation of exon 11. At the protein level, this splicing defect results in a 12-amino acid deletion (p.(Thr475_Val486del)), leading to the loss of two alpha-helices that form part of the alpha-helical repeat domain. This structural alteration is predicted to impair the functional integrity of the protein as illustrated in Figures 3B,C.

This variant was submitted to ClinVar and registered under accession number VCV003906956.1 on July 5, 2025.

## 4 Discussion

The *FOCAD* gene encodes a cytoplasmic protein that regulates focal adhesion assembly, microtubule dynamics and cell cycle progression. It has been implicated in tumor suppression, particularly in colorectal cancer and gliomas and contributes to epithelial homeostasis (9). Recent functional data also highlight its role in stabilizing SKIC2 and SKIC3, core components of the SKI complex involved in mRNA quality control. Disruption of this pathway in hepatocytes leads to the accumulation of aberrant transcripts, activation of inflammatory signaling pathways, hypoalbuminemia and enhanced secretion of CCL2—a cytokine that promotes fibrogenic responses. These mechanisms align *FOCAD*-associated liver disease with disorders of RNA surveillance, such as tricho-hepato-enteric syndrome (THES), though *FOCAD*-deficient individuals notably lack the skin features.

Recent studies also demonstrate that *FOCAD* plays a critical role in promoting cysteine deprivation-induced ferroptosis via activation of the FAK signaling pathway, enhancement of mitochondrial TCA cycle activity, and stimulation of Complex I in the electron transport chain. This places FOCAD at the interface between mitochondrial metabolism and regulated cell death, further aligning *FOCAD*-associated disease with ferroptosis-related pathomechanisms (10).

In 2022, Traspas et al. reported 14 individuals from 10 unrelated families diagnosed with a syndromic form of neonatal liver cirrhosis associated with biallelic variants in *FOCAD* (4). Across these cases, a total of 14 pathogenic or likely pathogenic variants were identified, including three nonsense variants (p.Arg863*, p.Arg195*, p.Gln154*), two frameshift variants (p.Trp893Leufs*32,* p.Leu1448Cysfs3), five splice-site variants, and one large genomic deletion of 164.8 kb affecting exons 14–37. All variants were predicted to result in complete or near-complete loss of functional protein. The clinical presentation was notably uniform, comprising hepatomegaly, micronodular cirrhosis, elevated transaminases, cholestasis, hypoalbuminemia and coagulopathy. Additional systemic findings included abdominal distension, diarrhea, feeding difficulties, umbilical and inguinoscrotal hernias, intrauterine growth restriction (IUGR), hematological abnormalities and in several cases, craniofacial features such as a triangular face, high forehead and plagiocephaly.

More recently, Raja et al. (2025) reported a second clinical case of *FOCAD* deficiency presenting as rapidly progressive neonatal liver cirrhosis in a preterm infant compound heterozygous for c.4435del (p.Lys1475Asnfs*) and an exon 6–7 deletion (11). The patient developed severe hepatic dysfunction characterized by hypoalbuminemia, coagulopathy, ascites, and portal hypertension, ultimately requiring liver transplantation at 3 months of age.

Here, we describe a patient with infantile liver cirrhosis harboring a previously unreported homozygous splice-site variant, NM_001375570.1:c.1455 + 1G > T. Functional RNA analysis demonstrated that this variant causes consistent skipping of 36 nucleotides within exon 11, resulting in an in-frame deletion of 12 amino acids (p.Thr475_Val486del) that affects two conserved *α*-helices within the protein’s repeat domain. Structural modeling predicts a destabilizing effect on domain integrity, providing strong mechanistic evidence supporting the pathogenicity of this splice-disrupting variant. Our findings demonstrate a loss-of-function effect consistent with previously described *FOCAD*-related cases.

Clinically, our patient exhibited classical hepatic manifestations, including impaired weight gain, hepatomegaly, elevated liver enzymes, cholestasis, thrombocytopenia, dyslipidemia, and coagulopathy—all features variably observed in previously reported patients by Traspas et al. (4). Specifically, hepatomegaly, elevated liver enzymes, and cholestasis were observed in 71.4, 71.4, and 35.7% of those cases, respectively (see Table 2). Additionally, our patient presented with intrauterine growth restriction (IUGR) and persistent diarrhea, features also noted in the majority of previously described individuals.

**Table 2:** Clinical comparison between the proband and previously published patients.

Clinical synopsis	Published cases (%) (4)	Our case
Hepatic abnormalities	100%	+
Hepatomegaly	71.4%	+
Cirrhosis (METAVIR 4)	71.4%	+
Elevated hepatic enzymes	71.4%	+
Cholestasis	35.7%	+
Elevated hepatic iron concentration	21.4%	−
Intrauterine growth restriction	64.3%	+
Diarrhea	57.1%	+
Platelet/coagulation abnormalities	50.0%	+
Cardiac abnormalities	42.9%	−
Supravalvular aortic stenosis	7.1%	−
Ventricular septal defect	7.1%	−
Craniofacial abnormalities	35.7%	+
Skin abnormalities	21.4%	−
Hair abnormalities	7.1%	+
Immune deficiency	7.1%	+
Another finding		
Unilateral inguinoscrotal hernia	ND	+
Microcephaly	−	+
Eyes abnormalities	−	+

In contrast to the craniofacial features described by Traspas et al.—limited to a triangular facial shape, high forehead, and plagiocephaly—our patient displayed a more distinctive and extensive pattern of craniofacial dysmorphism. These included a round face, prominent metopic ridge, narrow forehead, long and broad eyebrows, deeply set eyes, epicanthal folds, a low-hanging columella, broad philtrum, macrotia, and sparse, thin scalp hair. While craniofacial and hair abnormalities were reported in only 35.7 and 7.1% of published cases, respectively, several of the features observed in our patient—particularly microcephaly and ocular involvement—have not been previously associated with *FOCAD*-related disorders. These findings expand the phenotypic spectrum of *FOCAD*-associated liver disease and suggest the presence of additional, potentially population-specific dysmorphic features (Table 2).

To date, two patients with *FOCAD*-associated cirrhosis have undergone liver transplantation. The first case, involving a compound heterozygous state for a splice-site variant and a gross deletion, resulted in death 2 weeks postoperatively due to multiorgan failure (4). The second case involved successful liver transplantation at 3 months of age (11). In our study, we report long-term survival following successful liver transplantation in this condition. Over an almost two-year follow-up period, the patient demonstrated normalized liver function and age-appropriate developmental milestones, underscoring the therapeutic potential of transplantation in selected individuals.

In addition, our patient demonstrated immunological involvement in the form of neutropenia. Several mechanisms may underlie this manifestation. Loss of RNA quality control may lead to the accumulation of aberrant transcripts and activation of cellular stress pathways, including innate immune sensors, thereby disturbing hematopoietic homeostasis. A comparable mechanism is observed in trichohepatoenteric syndrome (THES), where defective RNA degradation due to disruption of the SKI complex results in immune deficiency, underscoring the potential importance of RNA surveillance in immune regulation. Impaired FOCAD function may similarly compromise granulopoiesis, leading to increased apoptosis or arrested maturation of neutrophil precursors. Furthermore, altered RNA metabolism could interfere with immune regulatory networks, contributing to abnormal cytokine signaling and exaggerated inflammatory responses, which in turn may promote peripheral neutropenia through enhanced consumption or aberrant trafficking of neutrophils. Impairment of RNA turnover in immune cells may also compromise adaptive immune responses, predisposing to broader immunological dysfunction. Collectively, these observations suggest that FOCAD deficiency may contribute to immune abnormalities through a multifactorial pathogenesis involving defective hematopoiesis, immune dysregulation and inflammation. Nevertheless, the precise mechanisms remain insufficiently characterized and require further investigation.

Our study demonstrates that *FOCAD*-associated cirrhosis encompasses a broad spectrum of clinical manifestations and highlights the urgent need to systematically collect clinical data and conduct further research. Continued characterization of affected individuals will be crucial not only for delineating novel disease mechanisms, particularly those underlying immunological complications, but also for identifying potential therapeutic targets and guiding the development of future treatment strategies.

## 5 Conclusion

In conclusion, this report expands the genotypic and phenotypic landscape of *FOCAD*-associated liver disease. We describe a novel biallelic splice-site variant with experimentally validated pathogenicity, identify distinctive craniofacial characteristics including microcephaly, and provide the first evidence of sustained clinical benefit following liver transplantation. These findings reinforce the importance of early molecular diagnosis, functional validation of candidate variants and longitudinal clinical monitoring. Further investigation is warranted to elucidate genotype–phenotype correlations, assess population-specific variability and develop targeted strategies for therapeutic intervention in this rare and emerging disorder.

## Data Availability

The original contributions presented in the study are included in the article/supplementary material, further inquiries can be directed to the corresponding authors.
